# Multifocal Abdominal Pyomyositis From Subcutaneous Dissemination in an Immunocompetent Patient

**DOI:** 10.7759/cureus.23766

**Published:** 2022-04-02

**Authors:** Guillermo Ropero-Luis, Marina López-Núñez, Clara Hidalgo-López

**Affiliations:** 1 Department of Internal Medicine, Hospital de la Serranía, Ronda, ESP

**Keywords:** intramuscular injections, abdominal wall infection, cellulitis, infectious myositis, pyomyositis

## Abstract

A 61-year-old woman presented to the emergency ward complaining of low back pain for a month. She had undergone several spinal surgeries and a right radical nephrectomy 30 years before. A few days earlier she was injected with an intramuscular painkiller in her right buttock. An abdominal CT scan revealed multiple abscesses in the psoas muscle and the right posterior abdominal wall, including cellulitis in the adjacent subcutaneous tissue and the injection site. A diagnosis of pyomyositis from subcutaneous dissemination was made, and intravenous cefazolin was initiated. After five days of favorable progress, treatment was switched to oral cefadroxil to complete four weeks, leading to full recovery.

## Introduction

Pyomyositis, also known as infectious myositis, is a suppurative infection of skeletal muscle that usually occurs by hematogenous spread. It is more common in the tropics, and mainly affects children aged two to five years and adults aged 20 to 45 years. Most cases of pyomyositis in temperate regions (such as Spain) occur in middle-aged adults who are immunocompromised or are associated with other serious underlying conditions [1]. We present an unusual case of pyomyositis in a 61-year-old immunocompetent woman. The underlying cause was subcutaneous dissemination of local cellulitis caused by intramuscular injection at the site of a previous surgery.

## Case presentation

A 61-year-old woman presented to the emergency ward complaining of pain in the right lumbar region for the last month. She had a history of chronic low back pain due to severe scoliosis, and had undergone several orthopedic surgeries 30 years before (spinal arthrodesis with autologous bone grafts harvested from her right iliac crest). She also suffered from a right staghorn calculus, which resulted in an open radical nephrectomy. A few days earlier she had been given an intramuscular analgesic injection in her right buttock. She developed right flank pain, malaise and feverish feeling over the following days.

**Figure 1:**
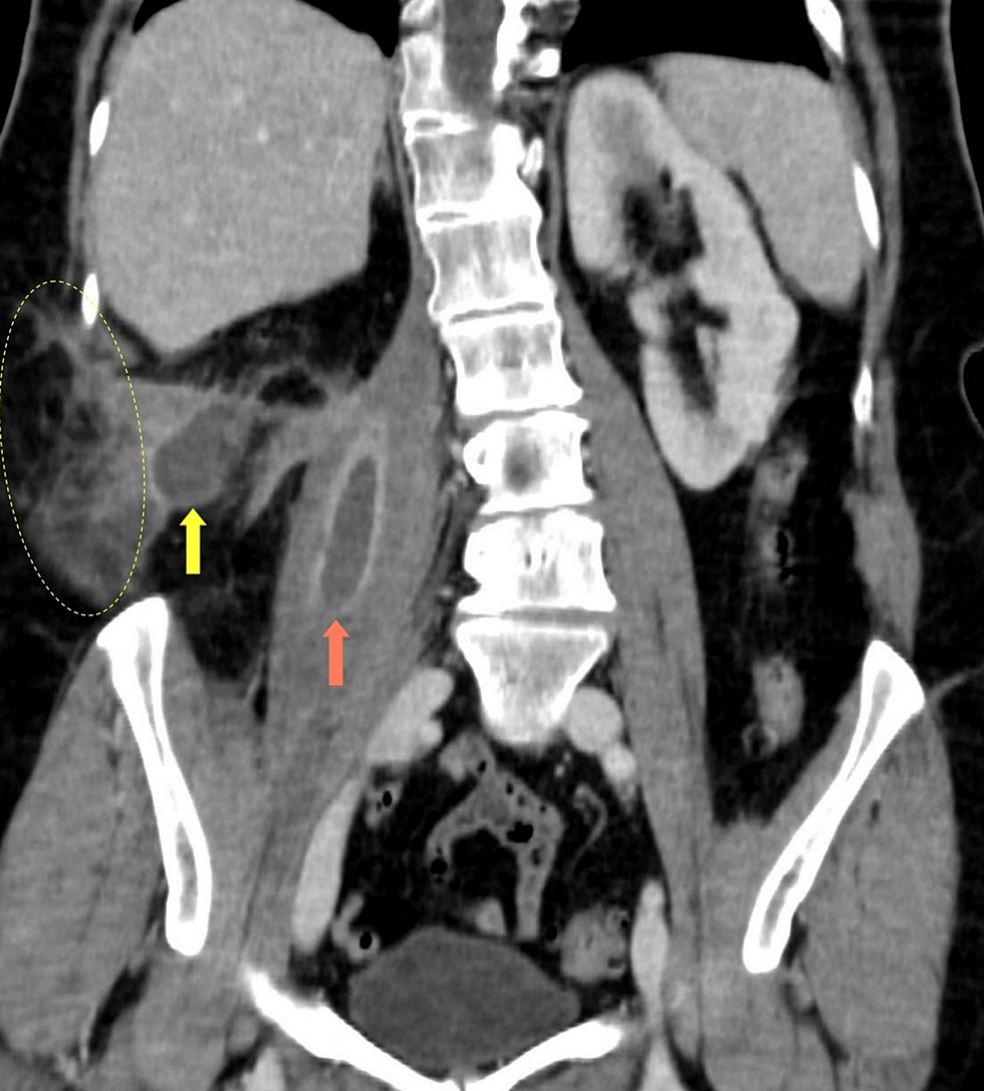
Coronal section of the abdominal CT scan performed on admission, showing abscesses in the psoas (orange arrow) and quadratus lumborum (yellow arrow) muscles, and local cellulitis adjacent to the latter (ellipse).

**Figure 2:**
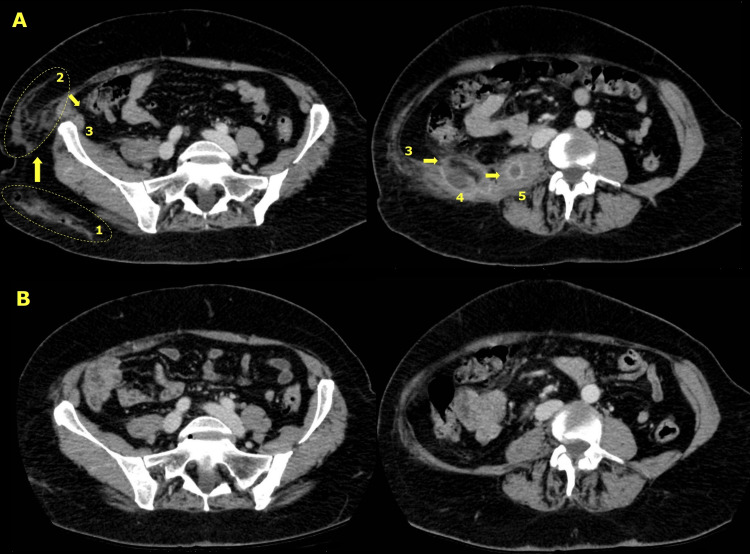
Axial sections of the abdominal CT scans performed on admission (top, A) and three months after discontinuing antibiotic therapy (bottom, B). Upper left, section showing cellulitis in the right buttock (ellipse 1) and adjacent (ellipse 2) to the right posterior abdominal wall (3). Upper right, section showing abscesses in the right quadratus lumborum (4) and psoas (5) muscles. The numbers and arrows indicate the likely route of dissemination. At the bottom, the same sections showing the full resolution of the lesions seen at the top.

Upon admission, the patient was hemodynamically stable and afebrile. Abdominal tenderness at right flank and right lumbar region was noted on the physical examination. Blood tests revealed mild leukocytosis (11,280/μL) with neutrophilia and marked C-reactive protein (CRP) elevation (113 mg/L; reference value below 5). A contrast-enhanced CT scan of the abdomen (Figures 1, 2A) showed destructuring of the right posterior abdominal wall (quadratus lumborum and oblique muscles), a nodular image measuring 54 mm by 30 mm by 34 mm in the quadratus lumborum muscle, local cellulitis in the adjacent subcutaneous tissue and the right buttock, enlargement of the right psoas muscle with a nodular hypodense image measuring 18 mm inside it, and inflammatory changes in the local retroperitoneum.

**Figure 3:**
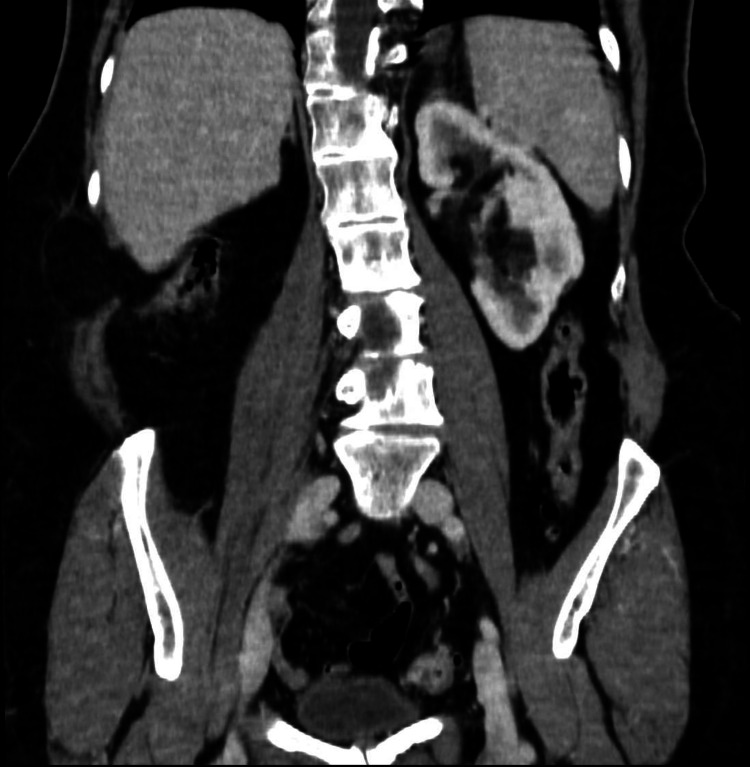
Coronal section of the abdominal CT scan obtained three months after discontinuing antibiotic therapy, showing complete resolution of the lesions seen in Figure 1.

Empirical antibiotic treatment with intravenous cefazolin (2 g three times a day) was initiated, based on the diagnosis of pyomyositis. The patient was admitted to the internal medicine ward. Blood cultures were taken after the first dose of antibiotic, and they came back negative. The patient’s clinical condition improved over the following days. Blood tests revealed normalisation of leukocytosis and CRP. A new CT scan after five days of treatment showed a positive radiological course and a decrease in the size of the abscesses, so image-guided aspiration was not attempted. We decided to switch the antibiotic treatment to oral cefadroxil (1 g two times a day), and she was discharged home. Laboratory tests and CT scan were repeated after three weeks of oral treatment, confirming the favourable course, so we decided to discontinue the antibiotics. A follow-up CT scan at three months showed complete resolution of the abscesses and the inflammatory changes in the subcutaneous tissue and the retroperitoneum (Figures 2B, 3). One year later, the patient remains asymptomatic with no signs of relapse.

## Discussion

Trauma, injection drug use, malnutrition and concurrent infections have been associated with pyomyositis [2]. *Staphylococcus aureus* is by far the most common cause, accounting for 75-90% of reported cases [3]. Blood cultures are usually negative. Treatment in the initial stages is antibiotics alone, directed against staphylococci and beta-hemolytic streptococci, although broader empirical antibiotic coverage is warranted in immunocompromised patients. The recommended regimen is three to four weeks of parenteral therapy, but should be tailored to the clinical course [4]. Patients with more advanced stages may require percutaneous or surgical drainage.

This case presents some peculiarities. The patient was above average age and had no significant comorbidities. We suspect that the pyomyositis arose from cellulitis caused by the intramuscular injection in the right buttock, which then propagated through the subcutaneous tissue to the posterior abdominal wall and the psoas muscle contiguously (Figure 2A). She had undergone several surgeries in this anatomical region, so the deep tissue planes of the surgical beds were compromised and facilitated dissemination. Given the favourable clinical course with first generation cephalosporin, we agreed with the patient to switch to oral therapy for discharge, with excellent outcome.

There are few studies reporting oral treatment for pyomyositis, most of them in children and adolescents. Published cases in adults (Table 1) include a 56-year-old female with an epidural abscess [5], a 51-year-old male [6] and a 24-year-old male [7] with acute myelogenous leukemia, an 18-year-old male with no apparent risk factors [8], and a 68-year-old male with chronic myeloid leukemia [9]. In comparison, our patient was switched to oral antibiotics earlier and the duration of treatment was shorter.

**Table 1:** Summary of studies reporting oral treatment for pyomyositis in adults. Ref.: Reference. M: Male. F: Female. IV: Intravenous.

Ref.	Age	Sex	Risk factors	Location	Etiology	Treatment
[5]	56	F	Former heroin addict by inhalation, smoker, moderate drinker	Left erector spinae muscle	S. aureus	IV Flucloxacillin 12 days + oral 6 weeks
[6]	51	M	Acute Myeloid Leukemia, chemotherapy	Left quadriceps femoris muscle	S. aureus	IV therapy 39 days + oral Levofloxacin 3 months
[7]	34	M	Acute Myeloid Leukemia	Right gluteal muscles	E. coli	IV Piperacillin-tazobactam + oral Levofloxacin 6 weeks
[8]	18	M	No (trauma during a rugby game?)	Left piriformis muscle	S. aureus	IV therapy 20 days + oral Flucloxacillin and Erythromycin 8 weeks
[9]	68	M	Chronic Myeloid Leukemia, imatinib, right hemicolectomy + ileocolostomy	Right thigh and iliopsoas muscles	Unknown	Oral Linezolid (unspecified treatment duration)

## Conclusions

Pyomyositis usually results from hematogenous spread, although in rare cases it can arise from local cellulitis. The infection can spread through the subcutaneous tissue, even in patients without significant comorbidities. Local skin and soft tissue infections in the site of previous surgery should be recognized as a risk factor for pyomyositis, so punctures in these areas are to be avoided. The recommended standard medical treatment is three to four weeks of intravenous antibiotics, but early switching to oral antibiotics appears to be a safe choice in uncomplicated patients, without the need for a prolonged course.
